# Effect of EMG-activated vibrotactile biofeedback on skill learning in children with genetic and acquired dystonia during point-to-point and cyclic task practice

**DOI:** 10.1186/s12984-025-01848-w

**Published:** 2025-12-31

**Authors:** Maral Kasiri, Emilia Ambrosini, Emilia Biffi, Shinichi Amano, Alessandra Pedrocchi, Nardo Nardocci, Elena Beretta, Giovanna Zorzi, Terence D. Sanger

**Affiliations:** 1 Department of Biomedical Engineering, University of California, Irvine, CA USA; 2 Department of Biomedical Engineering, University of Southern California, Los Angeles, CA USA; 3 Department of Electronics, Information and Bioengineering, Politecnico di Milano, Milan, Italy; 4 Scientific Institute, IRCCS E. Medea, Bosisio Parini, Lecco Italy; 5 U.O. Neuropsichiatria Infantile Fondazione IRCCS, Istituto Neurologico Carlo Besta, Milan, Italy; 6 Department of Neurology, Children’s Health Orange County, Orange, CA USA

**Keywords:** Dystonia, Motor control, Rehabilitation, Sensory deficit, Vibrotactile biofeedback

## Abstract

**Objective:**

Dystonia is a movement disorder that causes involuntary muscle contractions and abnormal movements. Often, repeated practice does not lead to motor improvement in children with acquired dystonia, likely because of sensory deficits, which may contribute to their impairment. Therefore, improvements in sensory function might improve motor performance. In this study, we propose that augmented vibrotactile biofeedback may improve motor learning in children with acquired dystonia but not in children with genetic dystonia who do not have associated sensory deficits.

**Design and participants:**

To test this hypothesis, we obtained muscle activity and kinematic recordings and computed outcome measures that represent motor skills during the practice of a point-to-point movement and trajectory-following task. We examined the effects of applying vibrotactile biofeedback on dystonic muscles in children who are typical and those with genetic and acquired dystonia.

**Results:**

The device significantly improved motor learning in children with acquired dystonia exclusively in the cyclic task, as evidenced by reduced error and an improved task correlation index (p < 0.01), whereas no significant effects were observed in the other groups.

**Conclusion:**

Our results show that the vibrotactile device potentially represents an effective method of motor improvement only for cyclic and smooth tasks but not for point-to-point tasks in children with acquired dystonia.

## Introduction

Sensory awareness is an effective way to improve motor performance [1–4]. Previous studies highlight the need to explore artificial sensory feedback as a noninvasive method for improving skill learning [5]. On the other hand, understanding how brain network disorders affect motor learning first requires an examination of the role of sensory deficits. Although artificial sensory augmentation benefits some adults in rehabilitation [6, 7], its effectiveness in children with dystonia remains unknown.

Dystonia is a movement disorder that can be genetic or acquired (due to another underlying disorder such as cerebral palsy (CP)) [8–10]. Although medication and surgery can be effective at ameliorating motor symptoms, noninvasive treatments remain valuable. Physical therapy is often ineffective at improving motor function in children with dystonia, possibly because of the “failure of motor learning” theory, which holds that without sufficient sensory feedback during movement, practice will fail to improve performance despite adequate repetition [11, 12].

Research has shown that sensory deficits appear in multiple forms of dystonia, including adult-onset focal hand dystonia and dyskinetic CP in children [5, 13], but not in genetic dystonia. Therefore, we propose that some children with dystonia fail to improve with practice because of their associated sensory deficits. If so, then biofeedback may enhance the sensory perception of movement and improve motor learning in children with dyskinetic CP who exhibit sensory deficits [5, 13–15]. Conversely, without sensory deficits, biofeedback likely has minimal effects. We examine whether an EMG-activated vibrotactile biofeedback (BF) device improves symptoms in children with acquired dystonia [8] by measuring their motor performance and comparing them with a group of children who are typical (control group) and a group of children with genetic dystonia.

Motor performance can be quantized by measuring movement accuracy, in various ways in different tasks [16]. For example, the speed‒accuracy trade-off of Fitts’ law explains the relationship between speed and endpoint accuracy during “fast” movement to a target [17–19]. One of the explanations for this phenomenon is that the trade-off represents compensation for signal-dependent noise; thus, moving slowly reduces noise and therefore increases accuracy [17–21]. Earlier studies have shown that the speed‒accuracy trade-off can be modified by practice and that the quantitative relationship between speed and accuracy may be an indicator of skill in some tasks. Improvements in this trade-off (achieving higher speed without loss of accuracy) reflects learning and increased motor efficacy [22–25]. Moreover, in earlier work, we showed that children with acquired dystonia are aware of their limitations and adjust their movements based on the large signal-dependent noise in their movement [16, 20, 22, 25, 26]. Because of the increased motor variability in children with dystonia, Fitts’ law is a good measure of the effect of dystonia on performance [21, 27, 28]. This is particularly relevant to children with acquired dystonia because improvement in the speed‒accuracy relationship could represent a reduction in a deficit associated with dystonia.

In this study, using Fitt’s law [17], we investigated whether augmented sensory feedback on a dystonic muscle helps children with acquired dystonia perform two different tasks. We asked whether biofeedback could increase the maximum speed for a given accuracy or improve accuracy at a given speed. We then evaluated performance using kinematic and muscle recruitment measures to identify any significant benefit from vibrotactile biofeedback.

## Methods

### Study design and participants

This multicenter crossover study involved 2 weeks of training with a wash-out period of 4 weeks. Weekly training was performed with or without the use of an EMG-based vibrotactile device. Three different clinical centers were involved in the studies: Neurological Institute IRCCS C. Besta, Milano, Italy; IRCCS Eugenio Medea, Bosisio Parini, Lecco, Italy; and Children’s Hospital, Los Angeles (CHLA), California, USA. The ethical committees of each center individually approved the protocol of the study (Neurological Institute IRCCS C. Besta: reference number 24 approved on 16-12-2015; IRCCS Eugenio Medea: reference number 054/14-CE approved on 01-04-2015; Children’s Hospital LA: reference number: CCI-11-00002 approved on 06-08-2011).

Three different groups of participants were recruited (56 total): 17 children and young adults with acquired dystonia due to cerebral palsy who were recruited and trained at IRCCS Eugenio Medea or at Children’s Hospital LA and University of Southern California (USC); 11 children and young adults diagnosed with genetic dystonia who were recruited at Neurological Institute IRCCS C. Besta and trained at Politecnico di Milano; and 28 children and young adults, as control group, who were recruited and trained at Children’s Hospital LA, USC, or at Politecnico di Milano. The inclusion criteria required participants to be of developmental age (6–20 years old), have no cognitive impairment preventing comprehension and communication, maintain stable drug therapy for dystonia without botulinum toxin in the dominant arm up to six months prior to enrollment, and be diagnosed by a pediatric movement disorder specialist using standard criteria [29]. They were eligible if they could perform the experimental tasks with at least one upper limb. All patients or their legal guardians provided signed informed consent for the Health Insurance Portability and Accountability Act (HIPAA) authorization for the use of protected health information and all the recorded data, in accordance with the Declaration of Helsinki, if they were recruited at the CHLA. All the patients or parents of participants (legal guardians) were recruited at IRCCS Eugeneo Medea and IRCSS C. Besta signed a written informed consent for the research and medical use of the recorded data and protected health information in accordance with the Declaration of Helsinki.

**Fig. 1:**
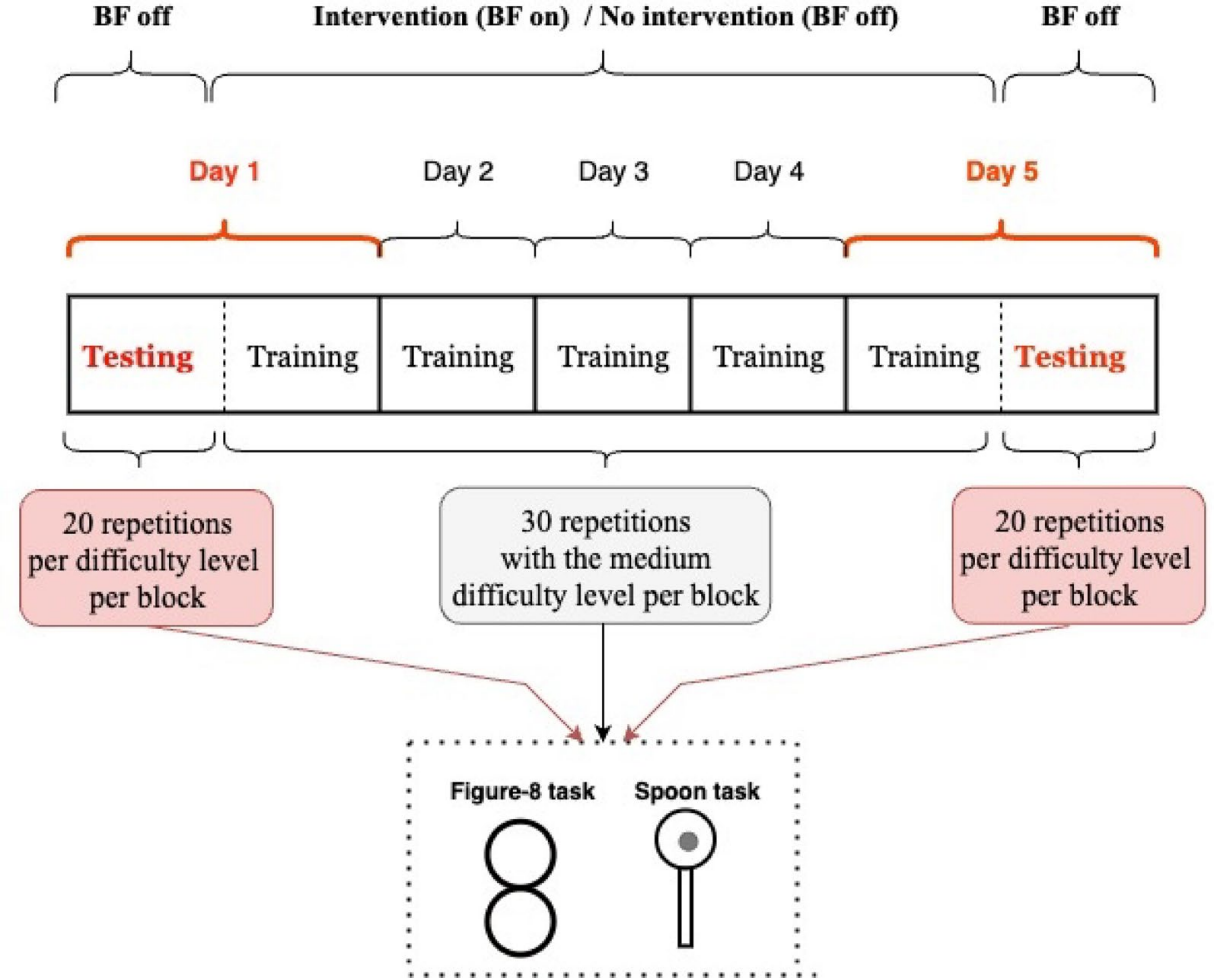
Experimental protocol sequences for one week. The study involved two training weeks separated by a four-week wash-out, with each week comprising five consecutive days of testing and training. Testing occurred on days 1 and 5 under three difficulty levels for each task, without biofeedback. Training was performed at medium difficulty on days 1–4 and before day 5 testing. For each task, a testing block included two trials of 10 repetitions (total of 20), and a training block included three trials of 10 repetitions (total of 30 per block)

This study included two separate training weeks, with a four-week wash-out period between them [27, 28]. Each week included five consecutive days of testing and training. In one week, training was conducted with the EMG-based vibrotactile biofeedback device; in the other, without the device. The order of these weeks was randomized to control for learning effects. After the four-week wash-out, participants returned to perform the same tasks for another five consecutive days, switching conditions (i.e., participants who trained with the device in the first week trained without it in the second, and vice versa). Each week began with a testing session on day 1 and ended with another on day 5, both performed under three different difficulty conditions for each task. No biofeedback device was used during the testing sessions. Training sessions took place immediately after the day 1 test, on days 2–4, and before the day 5 test [21, 27]. For each task and each difficulty level, each testing block consisted of two trials of 10 repetitions. For each task, each training block consisted of three trials of 10 repetitions, practiced at a medium difficulty level. See Fig. 1 for a schematic of the experimental protocol.

### Data recording

To evaluate the performance on testing days, kinematics of the upper arm and surface electromyography (EMG) from 8 upper limb muscles (e.g., flexor carpi radialis (FCR), extensor carpi ulnaris (ECU), and the biceps, triceps, supraspinatus, anterior, posterior, and lateral deltoids) were simultaneously recorded.

Kinematic data were collected using four Vicon Nexus 1.8.5 motion capture cameras (©Vicon Motion Systems Ltd, UK) with a sampling frequency of 100 Hz at USC, eight optoelectronic cameras from BTS Bioengineering with a sampling frequency of 60 Hz at IRCSS Medea, and POLARIS VICRA with a sampling frequency of 20 Hz at Politecnico di Milano. Passive reflective markers were placed on the upper extremity joints.

EMG signals were collected using a Biometrics 8-channel wireless EMG system, sampled at 1000 Hz at USC, BTS-Free EMG sampled at 1000 Hz at IRCCS Medea, and Porti 32 TMSi sampled at 2048 Hz at Politecnico di Milano. The maximum voluntary contraction (MVC) for all 8 muscles was recorded every day prior to the onset of the experiments. To record the MVC for each individual muscle, we positioned the participant’s arm in a manner that facilitated the isolation of that muscle. Initially, we recorded the baseline EMG, followed by instructing the participants to contract the isolated and stabilized muscle against resistance, maintaining maximal isometric contraction for 5 s.

**Fig. 2:**
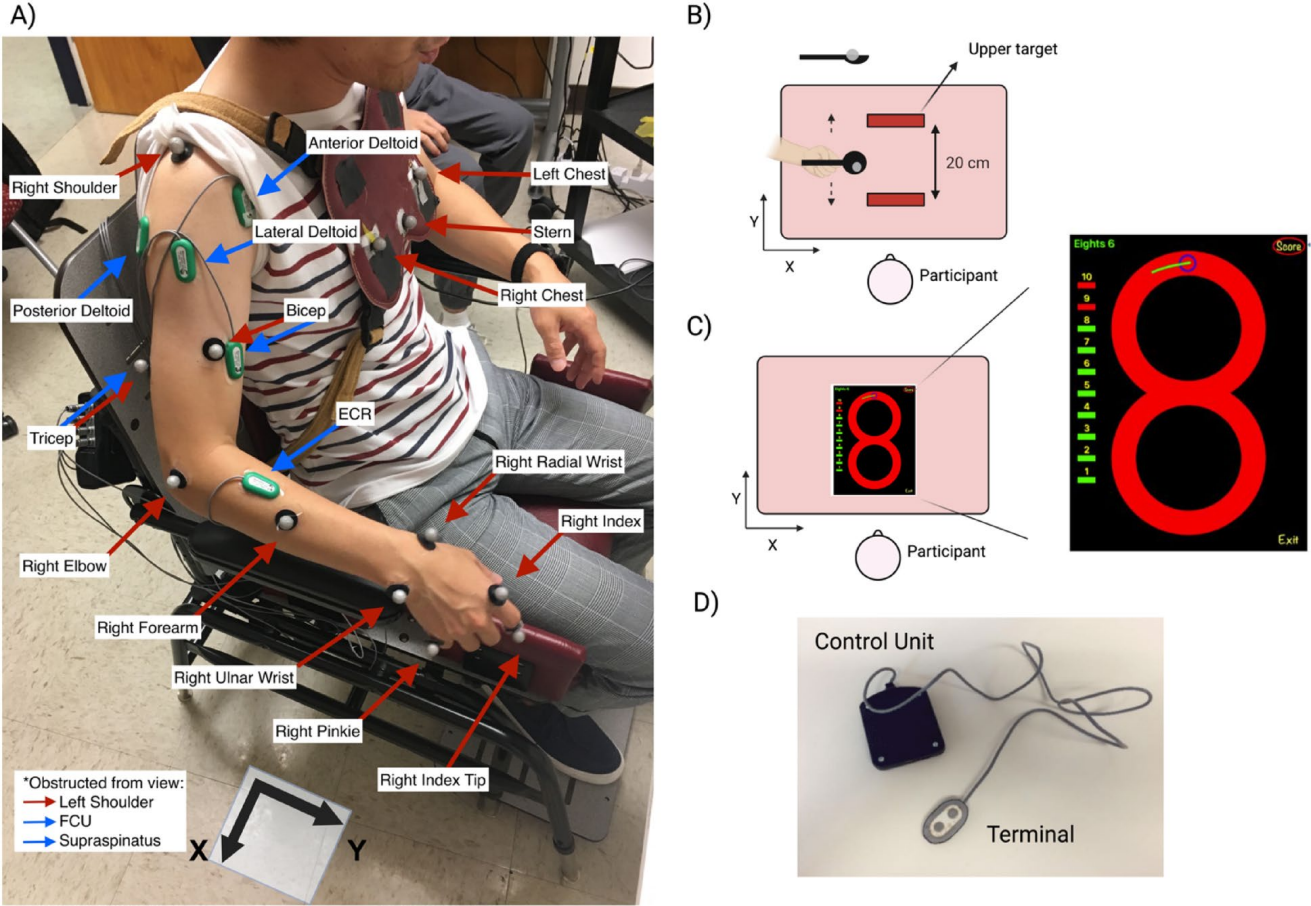
**A** Kinematic reflective marker and EMG sensor placement used at the USC. A total of 12 passive reflective markers were attached to the upper extremity joints and limbs to record the kinematic data. One additional marker was attached to the index finger in the figure-8 task to capture the finger trajectory. For the spoon task, one additional marker was attached to the spoon, and one sphere marker was used as the marble in the spoon (total of 14);** B** Spoon task setup: A board with two plastic blocks attached was placed on the table, with a vertical distance of 20 cm between the blocks. Participants began each trial at the upper target (the block furthest from them) with their arm fully extended. Each repetition consisted of a forward and backward movement starting and ending at the upper target;** C** Figure-8 task setup and iPad application: The scores displayed on the left side of the iPad app GUI indicated whether the participant successfully performed each repetition (green) or not (red). Participants began the task at the blue dot located above the figure-8 pattern, with their arm fully extended, and followed the red figure-8 trajectory line. They were required to adjust their speed so that they reached the top blue dot in sync with each metronome beat. The blue dot served as the fixed start point and did not move with the metronome;** D** The vibrotactile biofeedback device’s terminal head contained the filtering circuit, which adaptively filtered the EMG signal and calculated its envelope to estimate the output stimulation frequency, along with the vibrating motor attached to the target muscle. The control unit consisted of a microcontroller and a circuit for battery recharging [27]

The EMGs and motion capture system were synchronized by a trigger at the start of the movement. More accurate synchronization was performed with signal processing and cross-correlation of the signals. The setup and placement of the EMG and motion capture sensors are shown in Fig. 2A.

**Table 1:**
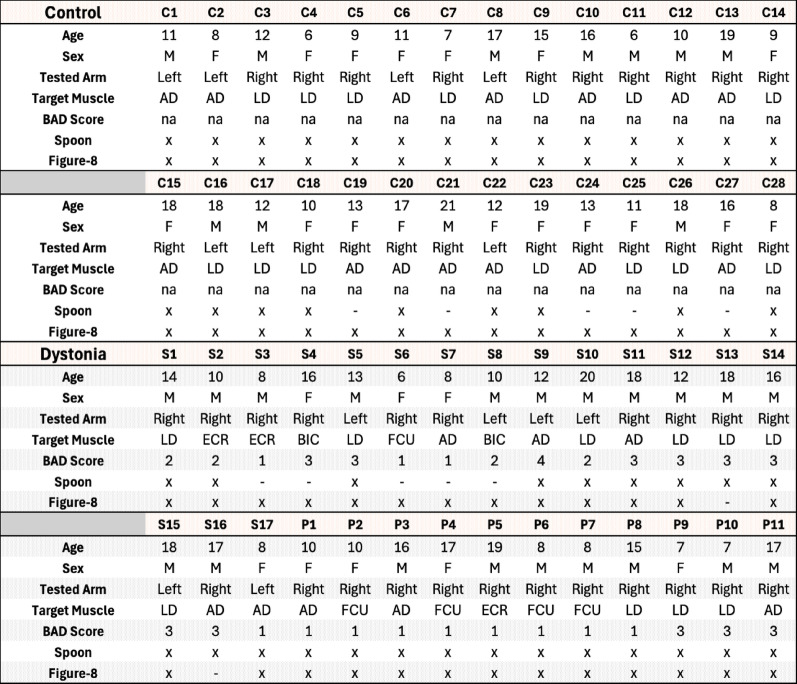
Participant demographics, targeted muscle for vibrotactile biofeedback, and Barry Albright dystonia (BAD) scale scores

The targeted muscle refers to the muscle on which biofeedback was applied during the experimental tasks. the BAD scale is a clinical measure of dystonia severity, with higher scores indicating more severe symptoms. An “x” in the spoon or figure-8 columns indicates that the participant was included in the analysis for that specific task. Data marked with “–” were excluded due to insufficient repetitions, data contamination, or to maintain age-matched balance between dystonia and control groups, ensuring comparable performance levels. *M* Male, *F* Female, *AD* anterior deltoid, *LD* lateral deltoid, *BIC* Biceps, *ECR* extensor carpi radialis, *FCU* flexor carpi Ulnaris, *Na* not applicable, the first letter of each subject ID stands for *C* Control, *S* Acquired, *P* genetic

### Experiments

#### Spoon task

This is a point-to-point, trajectory-constrained task, during which participants carried a reflective marble on a spoon between two targets without dropping it, which enforced a smooth velocity profile [21, 22]. Participants were seated upright in an adjustable chair, allowing them to modify the seat height and their distance from the table. Participants with severe dystonia used their own wheelchairs, which were positioned for optimal comfort and task performance. A board with two fixed targets was placed in front of the participants, flat on the table, such that they had to fully extend their arms to reach the farther target. Each target was bounded by plastic blocks spaced 20 cm apart from each other along the movement axis (y-axis) to limit forward and lateral acceleration. A reflective marker was attached to the spoon, and a separate spherical reflective marker served as the “marble”. Participants were asked to move the marble back and forth between the two targets as quickly as possible without dropping it. The setup [21, 22] is illustrated in Fig. 2B. Nine spoon sizes were used, and the index of difficulty (IoD) for each trial was calculated based on the spoon dimensions [21, 22, 26] as the ratio of the spoon diameter to its depth. Therefore, the index of difficulty is greater for shallower spoons with smaller diameters, as stabilization of marble in the spoon without dropping requires more precise attention to the smoothness of the trajectory. The details of the setup, spoon sizes, and IoDs are reported in our previous work [21, 22].

Prior to the experiment for each participant, performance was tested on a range of different spoon difficulties. Easy, medium, and difficult spoons were chosen for each participant. The difficult spoon was chosen as the largest Index of difficulty for which the participant could successfully transport the marble dropping it on fewer than 30% of the trials. The medium and easy spoons were the next 1 and 2 spoon difficulties below. On each trial, participants were asked to perform 10 forward and backward repetitions of the spoon task. Testing was performed with all three spoon sizes, with random orders. Training took place only with a medium spoon size for each participant (on days one through 5). No specific cost was associated with dropping the marble; however, if they dropped the marble three times or more out of ten repetitions of a trial, they were asked to redo the trial [21, 22]. Additionally, we asked the participants to perform the task without marble. This allowed us to measure the maximum unconstrained speed and represent the true Fitt’s law, which was based only on accuracy at the target endpoint without a constraint during the experiment, to assess the ceiling effect during practice.

#### Figure-8 task

This is a cyclic task constrained by movement speed (controlled by metronome), requiring participants to follow a figure-8 trajectory with their index finger [30, 31]. An iPad was placed, flat on the table, in front of the participant, and they were instructed to follow a figure-8 trajectory on the iPad (consisting of two circles 20 cm in diameter and 1 cm in width) with their index finger, as depicted in Fig. 2C. The 1 cm width represented the allowed borders, and participants were required to keep their movement within this path. The position of the iPad was adjusted so that the participants had to extend their arm fully to reach the top point of figure-8 with their index finger, maintaining contact between the finger and screen. Participants had to follow the trajectory clockwise or counterclockwise depending on the arm used. An additional marker for the tip of their index finger was used to capture the finger movement trajectory [31].

Prior to the experiment, performance was tested on a range of different speeds (10 to 45 bmp) controlled using a metronome. The fastest tempo was chosen as the most difficult task for which the participant could successfully follow figure-8 with fewer than 30% failure to follow the trajectory line. The medium and easy levels were the next lowest (5 and 10 bpm, respectively). Testing was performed at all three speeds with random ordering, but training was performed only at medium speed. On each trial, participants were asked to perform the figure-8 for 10 repetitions. Participants were encouraged to make continuous movements, not stopping at any point on the iPad. Therefore, the first 5 repetitions were performed with the metronome on. For the next 5 repetitions, we turned off the metronome, and they had to remember the speed of movement and continue the task, allowing for smoother and continuous movements, with small deviations from the metronome speed. The figure-8 application on the iPad could detect whether participants were moving out of the allowed borders and would mark that repetition as a failed one.

### Vibrotactile biofeedback device

The vibrotactile biofeedback device, shown in Fig. 2D, contains a vibration motor and active differential electrode head that records target muscle activity [27]. The vibration motor is at the head of the device, and feedback is applied directly at the site of muscle‒electrode contact for a clear and relevant stimulus. The vibration frequency ranged from 10 to 55 Hz and was modulated in real time based on EMG activity from the targeted muscle, such that higher EMG activity produced higher vibration frequency. The amplitude of stimulation was fixed at the device’s default output level, which is sufficient for clear tactile perception without causing discomfort. This electrode head is connected to a control unit that computes the amplitude of the recorded EMG signal through Bayesian estimation and controls the silent vibration motor with a rotation speed and amplitude proportional to the magnitude of the EMG. The processor and nonlinear filter in the device are designed to enable proportional biofeedback [27, 28]. In children with dystonia, each participant’s most affected muscle of dominant arm received vibrotactile feedback. For the control group, either the lateral deltoid (LD) or anterior deltoid (AD) of their dominant arm was randomly selected as the target (vibrated) muscle, as shown in Table 1.

### Data processing

**Fig. 3:**
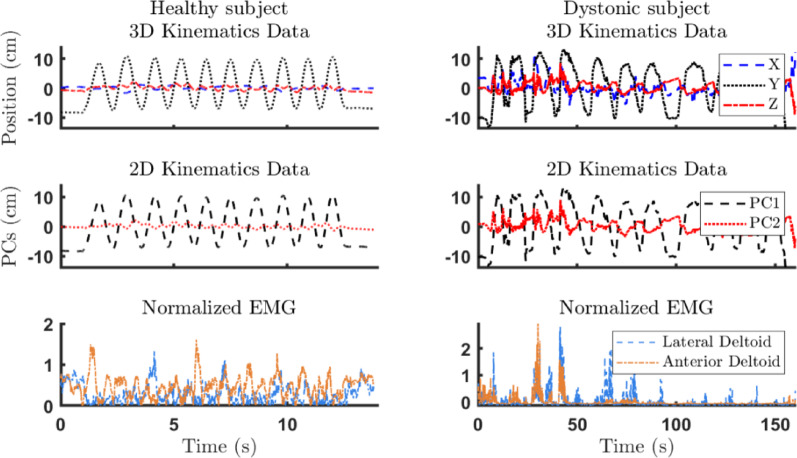
Samples of kinematic data and EMGs for a full-trial spoon task for a healthy (left) and a participant with dystonia (right); (top) 3D kinematic data recorded during the performance of the spoon task in the X, Y, and Z directions. (middle) First two principal components of X, Y, and Z recordings. These two PCs were used throughout the analysis. (bottom) Corresponding EMG recordings of the anterior and lateral deltoid

Data processing was conducted in MATLAB R2020a (MathWorks, Natick, MA, USA). All the EMG signals were bandpass filtered at 2–200 Hz. The MVCs were smoothed and rectified. For each muscle, the mean RMS value from a 1-s plateau during the highest MVC trial was used as the reference, and task EMG was divided by this value for normalization. Kinematic data were reconstructed and interpolated as needed in Cortex 5.5 (Motion Analysis Corp., Rohnert Park, CA, USA). After applying a 5 Hz low-pass filter to the kinematic data, we used principal component analysis (PCA) to extract 2D joint coordinates from the 3D data. Kinematic data and EMG signals were first resampled to a common sampling frequency of 1000 Hz to ensure temporal consistency across data streams. Initial alignment was based on the trigger signal recorded during the experiment; however, as the trigger provided only an approximate synchronization, further refinement was performed using cross-correlation analysis (“xcorr” function in MATLAB) between the EMG and kinematic signals to determine and correct for any time lag. This process enabled precise alignment of the two datasets. The synchronized data were then segmented into individual repetitions corresponding to the figure-8 or spoon tasks for subsequent analysis. The segments in which participants dropped the marble or lifted their fingers from the iPad were treated as outliers. The preprocessed EMG and kinematic data from a healthy participant and a participant with dystonia performing the spoon task are shown in Fig. 3. Finally, we calculated the average movement time in each repetition and then derived outcome measures for statistical analysis.

### Spoon task outcome measures

*Index of Performance (IP)* We fitted a regression line to the movement time (MT) and the Index of difficulty and calculated the index of performance (IP) as the inverse of the MT-Index of difficulty slope [17]. For each participant, we compared Day 1 and Day 5 IPs to determine whether improvement was greater during the “biofeedback week (BF on)” than during the “no-biofeedback week (BF off)” [17–19].

*Co-contraction index (CCI)* The co-contraction index is a useful parameter for evaluating performance, especially in point-to-point tasks. Muscle coactivation plays an important role in performing these tasks, as sudden changes in direction require accurate and timely activation of agonist–antagonist muscles [32, 33]. This measure for a pair of agonist–antagonists at each repetition is computed as follows [33, 34]:

1 $$ \begin{aligned} \:CCI = & mean\left( {\frac{{{\mathrm{min}}(EMG_{i} ,EMG_{j} )}}{{{\mathrm{max}}(EMG_{i} ,EMG_{j} )}}} \right) \\ & *\left( {{\mathrm{min}}\left( {EMG_{i} ,EMG_{j} } \right) + {\mathrm{max}}\left( {EMG_{i} ,EMG_{j} } \right)} \right) \\ \end{aligned} $$

where *CCI* is the co-contraction index, “*min*” is the minimum and “*max*” is the maximum of an EMG pair (*EMG*_*i*_ and *EMG*_*j*_) at an instant in time. In this equation, If both muscles contract strongly at the same time (high EMG values with only a small difference between them), then both terms in the Eq. [1) the mean of their ratio, and 2) the sum of the minimum and maximum EMG values] will be large, resulting in a high CCI. Conversely, if one muscle is active while the other is relaxed, the CCI will be low.

### Figure-8 task outcome measures

Time×Error: This measure, derived from Schmidt’s law, a variation of Fitt’s law, captures performance in open-loop motions within thin lines [35]. We can assume that the movement is cyclic; therefore, time in this formula is the time required to complete a single figure-8 repetition. In this experiment, the error is computed as follows [30, 35]:

2 $$\:Error=\:\sqrt{\frac{1}{N}}*\:\sum\:_{t=1}^{N}{d\left(t\right)}^{2}.\:\:\:\:\:\:\:\:\:\:\:$$

Here, N is the number of samples, and d(t) is given as follows:

3 $$\:d\left(t\right)=\left(\frac{\mathrm{min}\left({d}_{U}-{d}_{L}\right)-radius}{radius}\right)\:\:\:$$

where dU and dL are the distances between the position and the upper and lower circles, respectively. The smaller the time×error is, the better the performance. Therefore, it should decrease with skill learning and motor improvement [35]. We compared this measure in the figure-8 task with the index of performance (IP) in the spoon task for the final evaluations.

Task correlation index (TCI): The figure-8 task captures both task-relevant and task-irrelevant frequencies in the kinematic and EMG signals. Because the trajectory is symmetrical, an ideal performance would show the X frequency as double the Y frequency, corresponding to four x-direction crossings for every two y-direction crossings. This design separates task-irrelevant (possibly dystonic) frequencies from task-relevant ones. The index is computed as follows:

4 $$\:{TCI}_{i}=\left(\frac{{PSD}_{EMGi\:}\left|\:{f}_{x}+{PSD}_{EMGi\:}\right|{f}_{y}}{{PSD}_{EMGi\:}}\right).\:\:\:\:\:\:\:$$

Here, $\:{PSD}_{EMGi\:\:}$ is the total power of muscle i’s EMG, and $\:{\:PSD}_{EMGi\:}|\:{f}_{x}$ and $\:{PSD}_{EMGi\:}|\:{f}_{y}$are the peak powers in the X and Y directions (at the task frequencies), respectively. This index ranges from 0 to 1, with higher values indicating better performance [31].

**Fig. 4:**
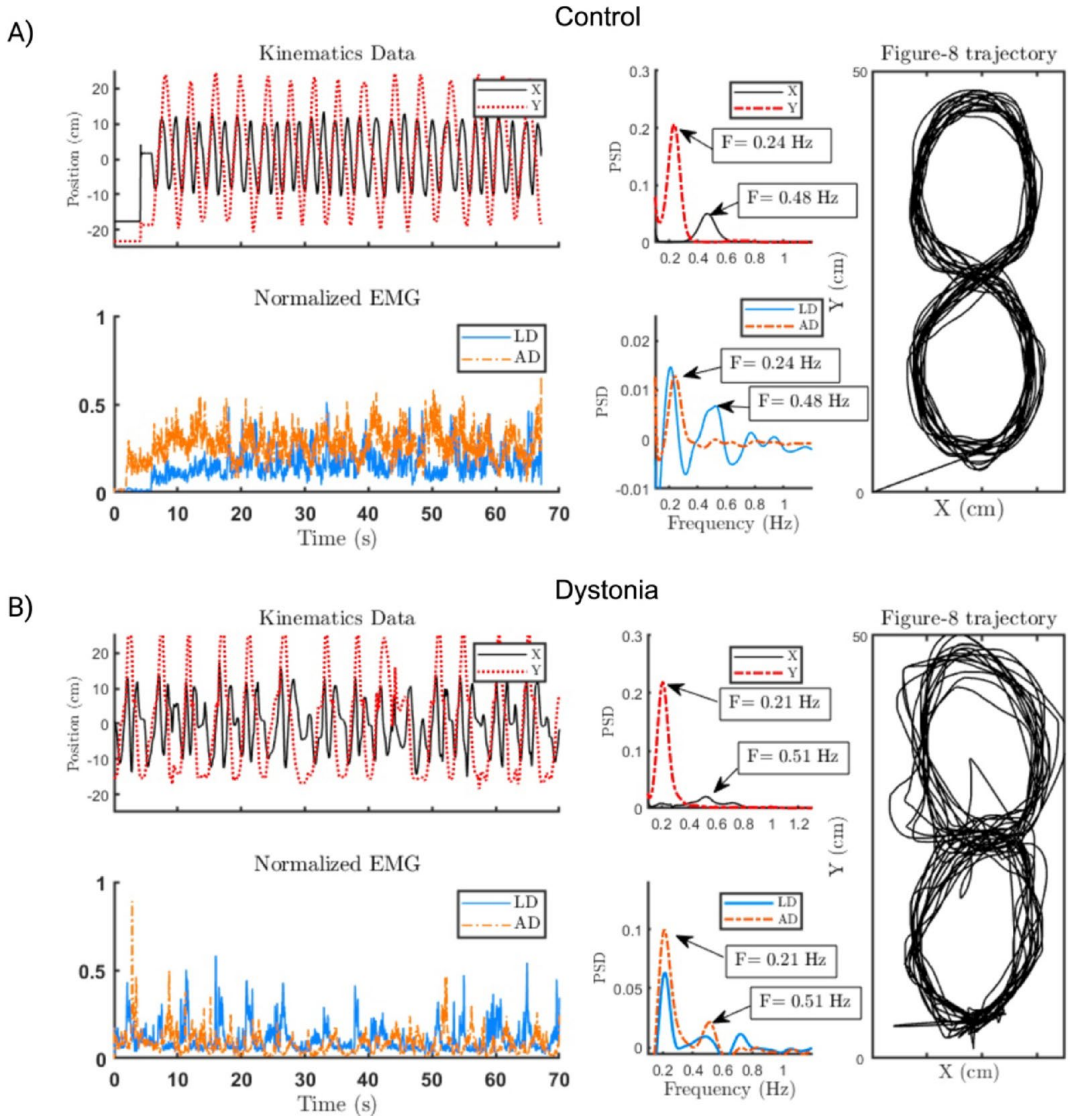
Recorded data during the performance of the figure-8 task for (**A**) a healthy and (**B**) a participant with dystonia. For each participant, (top) 2D kinematic data in the X and Y directions and their normalized spectrum, (bottom) the corresponding EMG recordings of the anterior and lateral deltoid and their spectrum, and (right) their corresponding movement trajectory are depicted. The X and Y spectra for the healthy participant show peaks at 0.48 and 0.24 Hz, respectively; one is exactly twice the other. The spectrum of the AD EMG only peaks at 0.24 Hz, contributing to movement only in the X direction, whereas the LD EMG spectrum peaks at both task frequencies, contributing to movement in both the X direction and the Y direction. For the participant with dystonia, the PSD of the LD and AD showed two peaks, only 0.51 and 0.21 Hz, respectively, contributing to the movements in both directions. Some overflow of activity in the LD is also observable at approximately 0.7 Hz

In Fig. 4, we present sample kinematic data, EMG spectra, and figure-8 trajectories from a healthy participant and a participant with acquired dystonia. Although the X and Y spectra of the participant with dystonia show distinct peaks, they are less sharp than those of healthy control are, and their frequencies are not precisely double, indicating movement overflow.

In other words, in the figure-8 task, the ideal performance is characterized by only two dominant frequencies (the two peaks shown in the PSDs in Fig. 4). The sharper these peaks, the better the muscle performance, as this indicates less overflow into irrelevant frequencies. A higher ratio of the power at these task-related peaks to the total power means that the muscle activity is more focused on the intended task rather than on noise or irrelevant movements.

### Statistical analysis

Statistical analyses of all the outcome measures were performed using the lme4 [36] and emmeans [37] packages in R-studio (R core team, 2021). A linear mixed effect model with repeated measures was employed to test the effect of practice on motor learning, with and without vibrotactile biofeedback, for all outcome measures. All p-values were adjusted for multiple comparisons using the Bonferroni correction to ensure reliability. For the spoon task outcome measures, we assessed the effects of the biofeedback device (on or off), group (i.e., genetic, acquired or healthy), testing day (i.e., Day 1 or Day 5), Index of difficulty, and their interactions, as independent variables (fixed effects), on all the outcome measures. In our model, random effects are intercepts for subjects, and by-subject random slopes are intercepts for the effect of the index of difficulty (Eq. 5). We used Kenward–Roger’s F test for pairwise comparisons and compared estimated marginal means of performance indices to assess the effect of biofeedback.

5 $${\text{Outcome measure}}\sim {\text{Testing day}} * {\mathrm{Group}} * {\mathrm{Week}} * {\text{IoD }}+{\text{ }}\left( {{\mathrm{IoD}}|{\mathrm{Subject}}} \right)$$

Figure-8 task measures were normalized for movement speed to remove the effect of task difficulty. The normalization for the speed-accuracy trade-off is done by using duration of repetition in the measure itself (time x error). The normalization of the TCI is done by calculating the normalized PSDs over the duration of each repetition. We then constructed a linear mixed-effects model with the same fixed and random effects used in the spoon task (Eq. 6). We used Kenward–Roger’s F test for and pairwise comparisons to assess the significance of each independent variable.

6 $$ {\text{Outcome measure }} \sim {\text{ Testing day }} * {\text{ Group }} * {\text{ Week + (1|Subject)}} $$

## Results

Fifty-six participants were recruited: 28 healthy controls, 17 with acquired dystonia due to cerebral palsy, and 11 with genetic dystonia. The characteristics and corresponding experimental details are presented in Table 1.

### Spoon task

**Fig. 5:**
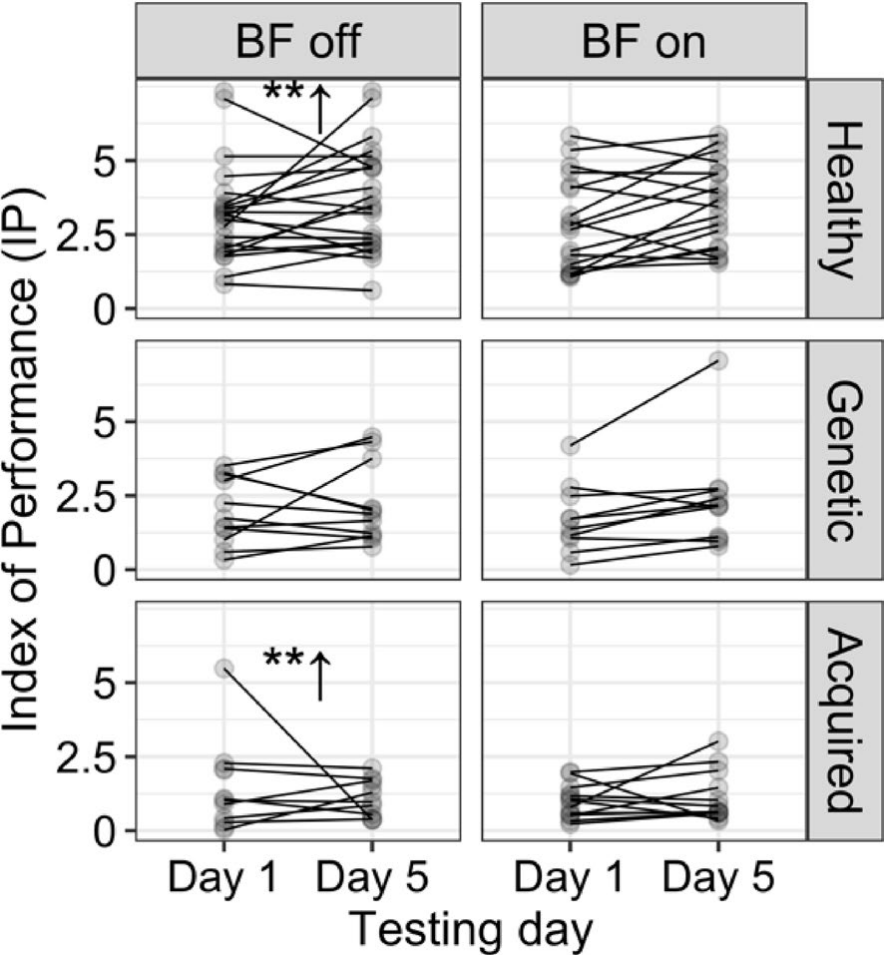
The index of performance (IP) for the spoon task showed no significant change or improvement with the biofeedback device. In contrast, learning occurred during the week without the device (BF-off) in both children with acquired dystonia and healthy participants (**p < 0.01). In other words, the IP increased significantly; however, the improvement from baseline after training with the device was not different from that observed during the week without the device

The regression lines of movement time versus the index of difficulty (R^2^ = 0.96) revealed a nonsignificant change in the index of performance for all children with genetic dystonia regardless of whether there was an intervention or no intervention during the practice. Although the participants with acquired dystonia and the control group moved faster after the practice without the device (indicated by a significant decrease in movement time, demonstrating learning *p* < 0.01) [22], the slopes of the regression lines remained unchanged compared to the week they trained with the biofeedback device, indicating that there was no significant improvement in index of performance. These results suggest that the children with acquired dystonia did not benefit from the vibrotactile biofeedback in the spoon task experiment, as shown in Fig. 5.

**Fig. 6:**
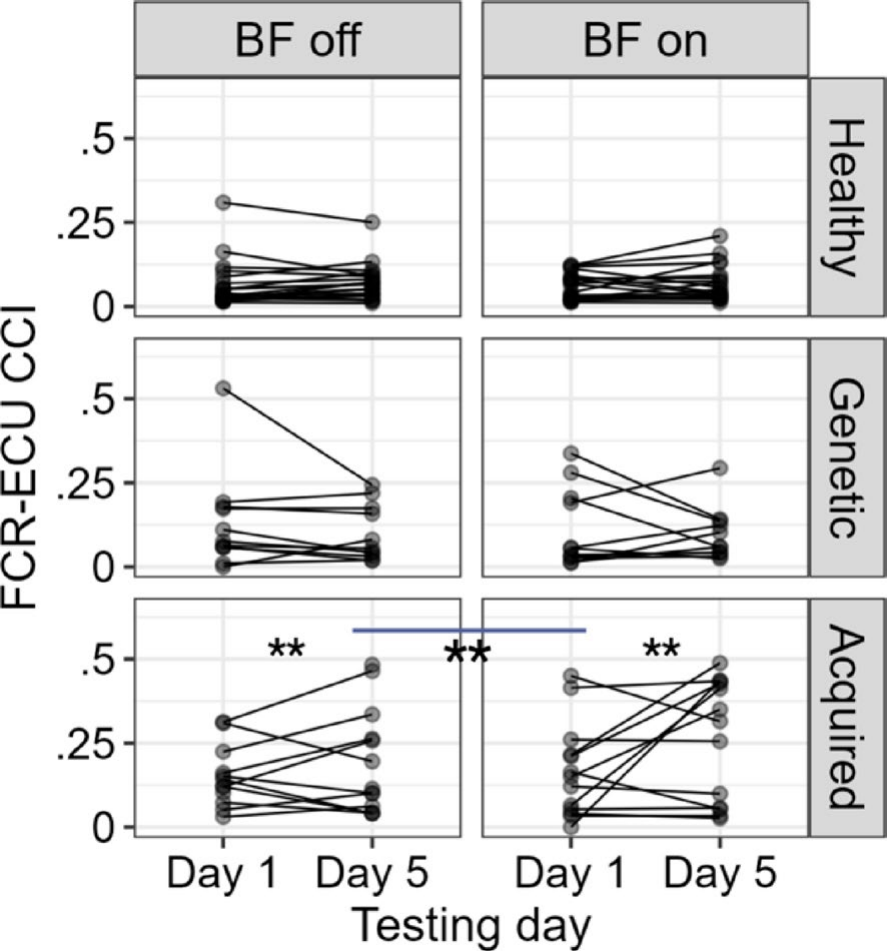
The vibrotactile biofeedback device significantly increased the FCU–ECR co-contraction index (**p < 0.01). Given the lack of improvement in spoon task performance with biofeedback, we examined whether the device adversely affected co-contraction indices (CCIs) for other muscle pairs. Analysis of FCR–ECU (R² = 0.72) showed that the device significantly increased the FCR–ECU CCI, with the effect size of 0.06 (mean BF-off vs. BF-on = 0.06, SE = 0.008, *p* < 0.01) exclusively in children with acquired dystonia

Owing to the ineffectiveness of the biofeedback device on spoon task performance, we evaluated the changes in the CCI of the FCR-ECU (R^2^ = 0.72), biceps-triceps (R^2^ = 0.64), and anterior-posterior deltoid (R^2^ = 0.72) to evaluate whether the biofeedback device affected the CCIs in an adverse way. The pairwise comparison of the estimated marginal means revealed that the device significantly increased the FCR-ECU CCI (*mean BF off vs. BF on = 0.06*,* SE = 0.008*,* p < 0.01)* only in children with acquired dystonia, as shown in Fig. 6. The effect of biofeedback on the biceps-triceps and AD-PD CCIs was negligible.

### Figure-8 task

**Fig. 7:**
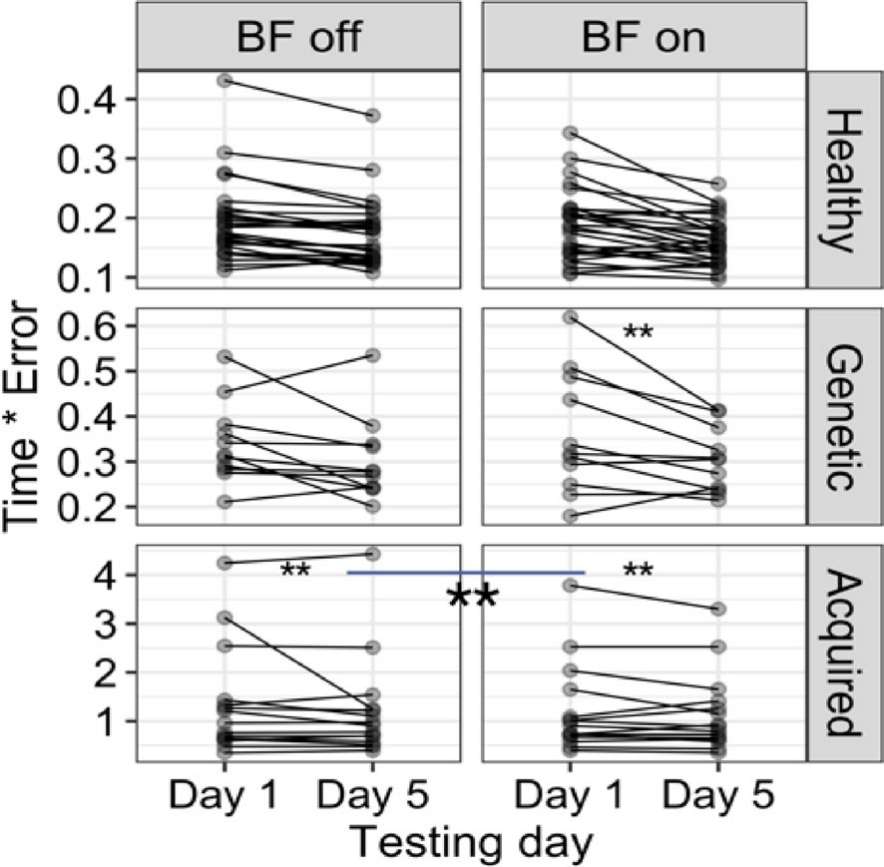
In children with acquired dystonia, the biofeedback device significantly reduced the time × error metric during the BF-on week compared with the BF-off week (shown with a line and ** connecting two blocks in children with acquired dystonia; *p* < 0.01), indicating improved performance. Here, the comparison reflects the change from baseline (Day 5 vs. Day 1) between the week with biofeedback and the week without biofeedback. The linear fit (R² = 0.88) and pairwise comparisons revealed a mean difference of 0.13 (SE = 0.02, *p* < 0.01, effect size = 0.13). No significant effect of the biofeedback device was observed in the healthy or genetic dystonia groups

**Fig. 8:**
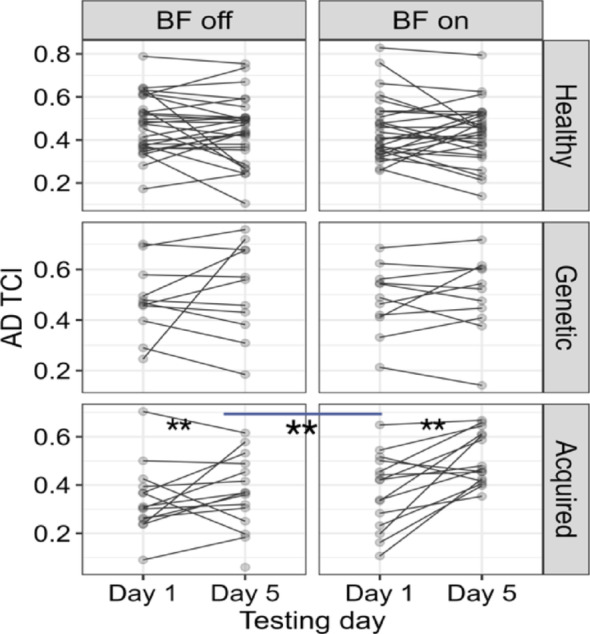
The vibrotactile biofeedback device improved the anterior deltoid task-correlation index (TCI) in children with acquired dystonia during the figure-8 task. Specifically, a significant increase (**p < 0.01; indicated by the line with **) in TCI was observed during the BF-on week compared to the BF-off week. The linear fit (R² = 0.57) showed that the anterior deltoid TCI increased significantly from Day 1 to Day 5 in the BF-on week (mean change = 0.11, SE = 0.02), and the improvement from baseline was significantly greater compared to the BF-off week (mean BF-off vs. BF-on difference = 0.05, SE = 0.01). No significant changes were found in the other muscles’ TCI

The linear fit (R^2^ = 0.88) and pairwise comparison for *time*×*error* revealed a significant decrease *(p < 0.01*) for the children with acquired dystonia with the biofeedback device on compared to the week that they practiced without the biofeedback, with effect size of 0.13 *(mean BF off vs. BF on = 0.13*,* SE = 0.02*,* p < 0.01*). Two other groups (healthy and genetic dystonia) did not show sensitivity to the biofeedback device (Fig. 7). The task correlation index for the anterior deltoid muscle (R^2^ = 0.57) increased significantly (p < 0.01) for the children with acquired dystonia while they performed the figure-8 task with the biofeedback device *(mean Day 1 vs. Day 5*,* BF = 0.11*,* SE = 0.02; mean BF off vs. BF on = 0.05*,* SE = 0.01)*, as shown in Fig. 8. However, this measure did not change significantly in the other muscles.

## Discussion

In this study, we hypothesized that the vibrotactile device would be effective at improving skill learning in children with acquired dystonia because of their sensory deficit. To test our hypothesis, we asked the participants to perform two tasks that were different in nature, following a specific protocol: the spoon task and the figure-8 task. In the spoon task, there is a smoothness constraint (“do not drop the marble”), and participants are asked to move as fast as possible [21]. In the figure-8 task, there is a time constraint indicated by a metronome, and participants are asked to follow the trajectory as accurately as possible [30]. By varying task difficulty, participants could choose their velocity profile in the spoon task [22] and their accuracy in the figure-8 task. If they learned an improved speed‒accuracy relationship, represented by the explained outcome measures, both tasks would show improved performance.

Our results suggest that this hypothesis is only partially correct. In other words, our results show that the vibrotactile biofeedback device improved motor learning in the figure-8 task (continuous task) but not in the spoon task (point-to-point task), indicating that children with acquired dystonia learned the figure-8 task significantly better when they practiced with the biofeedback device. This device seemed to decrease the movement variation in children with acquired dystonia, which could explain why we observed an increased task correlation index of the anterior deltoid (task-relevant muscle) in this group. On the other hand, this device does not seem to be very effective at improving the ability to learn the spoon task. Nonetheless, it was observed that learning took place during the week without any intervention (BF off), as reported in our previous study [22] These results could be explained by the different natures of the two experimental tasks. The spoon task places spatial accuracy constraints only on the endpoints of the movements, thereby emphasizing the importance of the feedforward control, whereas the figure-8 task requires ongoing [visual] feedback control due to the constraints on the fingertip path throughout the action. Previous studies have shown that children with dystonia fail in the timing of co-contraction due to the involuntary activation of an antagonist muscle during movement [32, 34, 38], leading to increased co-contraction during movement. Therefore, the lack of motor improvement when practicing the spoon task with biofeedback may be explained by the additional increase in the co-contraction index induced by the vibrotactile biofeedback, compounding their already heightened co-contraction. Future studies could investigate whether changes in muscle co-contraction patterns are necessary for the treatment to be effective in the spoon task. This change may be inconsistent among the participants, as each has a different baseline, adopts a different approach, and their dystonia manifests on different muscles. Another possibility is that the marble is effectively a biofeedback signal for movement, and this may have been more important in performance (drawing the attention to perform the task without dropping the marble) than the muscle EMG biofeedback, effectively drowning out any possible effect [38]. One other possibility is that the vibrotactile biofeedback improves only the figure-8 task performance because that task relies more on feedback control, which the biofeedback device essentially provides.

In this study, biofeedback enhanced performance in the figure-8 task but did not improve performance in the spoon task, indicating that mere practice is not adequate for achieving peak performance. The results for genetic dystonia revealed a lack of improvement in either task. Although this could occur because of multiple mechanisms, it is consistent with the hypothesis that biofeedback will improve movement only when a sensory deficit is at least partly responsible for poor movement and skill learning. This finding supports our initial hypothesis that acquired dystonia deficits might be partially due to learning failures stemming from sensory deficits. This proposition highlights the potential for noninvasive treatments for acquired dystonia that focus on augmenting sensation.

### Limitations

This study has several limitations that should be considered when interpreting the findings. First, direct quantitative sensory function assessments were not systematically collected for all participants, and thus we cannot directly correlate sensory deficits with the observed outcomes. Our hypothesis was instead based on existing evidence that secondary dystonia, particularly dyskinetic cerebral palsy, is commonly associated with sensory deficits [13]; however, we are not making direct conclusions about sensory status in this cohort. Second, data on the timing of brain injury, sensory deficit magnitude, and motor impairment severity were not consistently available and therefore were not included in the statistical model. To mitigate the influence of unmeasured parameters, we used linear mixed-effects models to account for subject-level variability. Third, although several meetings were held to standardize experimental protocols across testing sites, no formal validation study was conducted to confirm procedural equivalence; nevertheless, potential site effects are likely minimal because each participant’s performance was compared to their own baseline and data were normalized accordingly. Fourth, vibrotactile stimulation parameters were selected to provide consciously perceptible, EMG-modulated feedback without causing discomfort, but the effect of varying these parameters (e.g., amplitude, frequency range, mapping strategy) was not systematically studied, and should be investigated in future work [2, 6, 14, 32]. Finally, some participants exhibited increased co-contraction in certain conditions, highlighting a need for further optimization to avoid unintended muscle activation.

## Data Availability

The data and codes are available upon request.
